# Performance of thigh-mounted triaxial accelerometer algorithms in objective quantification of sedentary behaviour and physical activity in older adults

**DOI:** 10.1371/journal.pone.0188215

**Published:** 2017-11-20

**Authors:** Jorgen A. Wullems, Sabine M. P. Verschueren, Hans Degens, Christopher I. Morse, Gladys L. Onambélé

**Affiliations:** 1 Health, Exercise and Active Living Research Centre, Department of Exercise and Sport Science, Manchester Metropolitan University, Crewe, United Kingdom; 2 Musculoskeletal rehabilitation research group, Department of Rehabilitation Sciences, KU Leuven, Belgium; 3 School of Healthcare Science, Manchester Metropolitan University, Manchester, United Kingdom; 4 Lithuanian Sports University, Kaunas, Lithuania; Vanderbilt University, UNITED STATES

## Abstract

Accurate monitoring of sedentary behaviour and physical activity is key to investigate their exact role in healthy ageing. To date, accelerometers using cut-off point models are most preferred for this, however, machine learning seems a highly promising future alternative. Hence, the current study compared between cut-off point and machine learning algorithms, for optimal quantification of sedentary behaviour and physical activity intensities in the elderly. Thus, in a heterogeneous sample of forty participants (aged ≥60 years, 50% female) energy expenditure during laboratory-based activities (ranging from sedentary behaviour through to moderate-to-vigorous physical activity) was estimated by indirect calorimetry, whilst wearing triaxial thigh-mounted accelerometers. Three cut-off point algorithms and a Random Forest machine learning model were developed and cross-validated using the collected data. Detailed analyses were performed to check algorithm robustness, and examine and benchmark both overall and participant-specific balanced accuracies. This revealed that the four models can at least be used to confidently monitor sedentary behaviour and moderate-to-vigorous physical activity. Nevertheless, the machine learning algorithm outperformed the cut-off point models by being robust for all individual’s physiological and non-physiological characteristics and showing more performance of an acceptable level over the whole range of physical activity intensities. Therefore, we propose that Random Forest machine learning may be optimal for objective assessment of sedentary behaviour and physical activity in older adults using thigh-mounted triaxial accelerometry.

## Introduction

Ageing is associated with a decline in physical function and recent evidence not only suggests that this is largely attributable to increased sedentary behaviour (SB) in old age, but also states that breaking prolonged SB by carrying out physical activity (PA) of at least light-intensity may prove to be a promising counteraction strategy [1]. It is surprising that though most elderly exhibit high SB and low PA levels, leading to deleterious health outcomes, strategies to minimise poor lifestyle choices in this age group has only received relatively little scientific attention [1–3]. Ahead of this however, studies must first focus on improving the accuracy and validity of activity monitoring in older adults [4,5]. To evaluate the exact health effects of SB and PA, including their role in healthy ageing, it is important to accurately and objectively monitor these aspects of habitual mobility or lack thereof [6]. Motion-sensing technologies using accelerometers are typically used in mobility monitoring since they are assumed to be objective, and measurements can be carried out over a number of days [6–11].

The concept of accelerometry to assess SB and PA is derived from Newton’s Second Law, which gives the interaction between force, mass and acceleration by the formula: force = mass * acceleration [12]. In the context of human movement, this formula can be expressed as: an activity is characterised by moving a mass (i.e. body (segment)) at changing velocity over time (= acceleration). This acceleration results from forces generated by (and on) the muscles at the expense of energy [10]. Several studies have shown positive linear relationships between energy expenditure (EE) and movement acceleration in people of different ages, while performing activities under standardised test conditions with the accelerometer close to the centre of mass [13–18]. This allows EE to be estimated from acceleration signals and the classification of habitual daily activity as sedentary, light and moderate-to-vigorous, by using, until recently, cut-off point models. To illustrate this, when presenting the amount of movement acceleration as counts per minute, these models will classify an outcome of <100 as sedentary, 100–1951 as light and ≥1952 as moderate-to-vigorous [6].

However, with the preferred accelerometer mounting location shifting away from centre of mass sites such as the hip or waist [19–21], towards wrist-worn devices for the most part, the premise of a linear relationship between EE and movement acceleration and thus, the use of cut-off point models has become questionable. This commercially-led shift forces researchers to focus on posture detection only (i.e. the ‘Sedentary Sphere’ [22]) or to start looking into other, more sophisticated and complex, methods to analyse acceleration signals by e.g. machine learning [2,23,24]. Machine learning is already used for activity recognition and has only recently been explored in PA research [2,24]. By focusing on patterns and regularities, pattern recognition for example, can handle complex and non-linear data [6,25,26], potentially providing opportunities for SB and PA research [27].

Although some experts have advised to stop developing cut-off point algorithms and start using machine learning [4,28], to date the use of cut-off points remains preferred for intensity classification [29]. One reason to continue using cut-off point models lies in the complex nature of machine learning, and the ease to understand and widespread adoption of cut-off points [30]. Although proprietary cut-off points are not necessarily well understood either, the desire to compare results with previous cut-off point-based studies could be another reason. Notwithstanding, studies have already shown machine learning to outperform traditional cut-off point algorithms for activity recognition not only in healthy adults, but also in niche populations such as the young or the overweight/obese [6,27]. However, validation of machine learning needs to be confirmed for all intended end-users/study populations, e.g. the elderly, prior to general adoption [10]. Rosenberg et al. [31] recently showed high levels of accuracy and concurrent validity using Random Forest classifiers in older women.

The decision of researchers to choose a simpler, but less accurate method over a more challenging and accurate one for activity intensity classification can possibly be justified when using thigh-mounted triaxial accelerometry. Since the thigh is relatively close to the centre of mass, cut-off point models might still be valid in this situation, especially when adding posture detection to these models, which then enables distinguishing between sedentary activity and standing for instance. Whilst the activPAL inclinometer is a good example of a valid thigh-mounted activity monitor [20,22], it uses black-boxed proprietary algorithms, thereby hampering progress in thigh-mounted accelerometer algorithm development. To date, cut-off point models for thigh-mounted accelerometers are understudied, hence further investigation and detailed comparison with machine learning is needed.

All algorithms require value calibration and the eventual utility of an algorithm depends on the specific activities and intensities included in the calibration study [30]. To ensure high accuracy of the algorithm in the general population, it is recommended to perform the calibration on a heterogeneous sample, matching the population of interest, and including a broad range of common activities ranging from sedentary to vigorous intensity [4,24,30,32]. Algorithm performance is generally expressed in terms of overall accuracy and when it reaches ≥80% for example, an algorithm is deemed acceptable [2]. However, even in possession of the overall (i.e. group) accuracy, algorithm performance on an individual (i.e. single end-user) level, remains unknown. Theoretically, performance can be unacceptable in some individuals where algorithm robustness is lacking. If algorithm inaccuracy disproportionately affects some demographic groups over others, it may lead to misinterpretation of associations between either SB or PA and health. Thus, it is important to check robustness and benchmark end-user-specific performance of accelerometer algorithms developed on heterogeneous pooled-data sets prior to applying them to daily-life data. To date, evidence regarding this type of triangulation is sparse.

The main aim of the present study was to compare between traditional cut-off points and machine learning, for the provision of the best performing algorithm to classify SB and PA in a heterogeneous population of older adults using thigh-mounted triaxial accelerometry. It was hypothesised that machine learning outperforms cut-off point based algorithms through being robust for individual’s physiological and non-physiological characteristics, more accurate and showing acceptable accuracies for all activity intensities. To test this hypothesis, this paper 1) examines overall balanced accuracy and robustness of four heterogeneous pooled-data algorithms, 2) compares participant-specific balanced accuracies between all four algorithms, and 3) benchmarks both overall and participant-specific balanced accuracies of the algorithms.

## Materials and methods

### Participants

Forty healthy older adults (73.5 (6.3) years; 50% female) participated in this study (Table 1). Participants were excluded if they were: <60 years of age, terminally ill or receiving cancer treatment, diabetic, suffered from any central nervous system disease or condition, had a heart attack in the past 12 months or any currently unstable cardiovascular condition, had any pulmonary disease or condition that did not allow expired gas sampling, recently (within the past three months) injured or had surgery on either of their lower limbs, were not independently mobile or at least not able to complete a laboratory-based activity protocol without a (walking) aid, had been advised by their physician not to take on any physical activity or exercise, or were not competent to make an informed decision about study participation.

**Table 1:** Study sample characteristics.

Age (years)	73.5 (6.3)	
Sex	20 Female	20 Male
Body mass (kg)	72.2 (13.7)	
Body height (m)	1.67 (0.10)	
BMI (kg∙m^-2^)	25.6 (4.3)	
Prandial state	20 Fasting	20 Non-fasting
REE_fasting_ (VO_2_ ml∙kg^-1^∙min^-1^)	2.82 (1.00)	
Prosthetic lower limb joints	2 Yes	38 No
Cardiovascular medication	20 Yes	20 No
Physical fitness level_no cardiovascular meds_	9 Less than good	11 Good or better
Preferred walking speed (km∙h^-1^)_no prosthetic lower limb joints_	3.7 (1.0)	
Falls risk	32 Low	8 Medium or high

Values represent arithmetic mean (SD) when normally distributed data, else median (IQR).

SD, standard deviation; IQR, interquartile range; BMI, body mass index; REE, resting energy expenditure; VO_2_, oxygen consumption.

This study was approved by the local ethics committee of the Manchester Metropolitan University, UK. All participants gave written informed consent prior to their participation in this study.

### Baseline characteristics

From each participant, the following baseline characteristics were recorded: age, sex, body mass, body height, body mass index (BMI), prandial state, resting energy expenditure (REE), presence of prosthetic lower limb joints, use of heart rate controlling medication, physical fitness level, preferred walking speed and risk of falling (Table 1). Age (years), sex (female/male), prandial state (fasting/non-fasting), presence of prosthetic lower limb joints (yes/no) and use of cardiovascular (heart rate controlling) medication (yes/no) was determined through a health questionnaire or orally on the day of testing. Body mass was assessed in kilograms using a digital body mass scale (Seca GmbH & Co. KG., Hamburg, Germany) and body height was measured in centimetres using a stadiometer (Holtain Ltd., Crymych, UK). Both measures were determined up to the closest decimal with the participant barefoot and wearing light clothing only. The body mass index (BMI) was calculated by dividing body mass by squared body height (kg∙m^-2^). REE was estimated by assessing oxygen consumption (VO_2_) (ml∙kg^-1^∙min^-1^; STPD conditions: standard temperature and dry gas at standard barometric pressure) while sitting quietly on a chair for four minutes, together with resting heart rate (beats per minute). Both REE and resting heart rate were expressed as the arithmetic mean of the readings taken during the third and fourth minute of sitting. To increase the accuracy of REE baseline estimates, only data from fasted participants were used. Since resting heart rate served to estimate baseline physical fitness levels, participants who were on heart rate controlling medication were not taken into account. Classification of the physical fitness levels was done using a standard resting heart rate table [33]. Preferred walking speed (km∙h^-1^) was based on the self-selected speed during treadmill walking in participants without prosthetic lower limb joints. Risk of falling (low/medium/high) was determined using the falls risk assessment tool (FRAT) [34].

### Instrumentation

During the laboratory-based activity protocol participants were equipped with different instruments. First, two GENEActiv Original triaxial accelerometers (Activinsights Ltd., Kimbolton, UK) with range ±8 g (1 g = 9.81 m∙s^-2^) and weighing 16 grams each, were fitted bilaterally on the anterior mid-thigh (at 50% of the distance between trochanter major and lateral femur epicondyle). Both accelerometers were mounted using Tegaderm^™^ transparent film dressing (3M Health Care, St. Paul, MN, USA) and set at a sample rate of 60 Hz. This frequency respects the Nyquist-Shannon sampling theorem, which states that the sample frequency should at least be twice the maximum frequency at which sampling is required. Since essentially all human body movement occurs below 20 Hz, the sampling rate should be ≥40 Hz [35,36]. Orientation of the accelerometer axes during standing was: X = mediolateral, Y = vertical and Z = anteroposterior. The devices were used as calibrated by the manufacturer. Next, participants wore a Polar T31 chest belt to monitor heart rate, which would then remain in place for the entirety of the test protocol (Polar Electro Oy, Kempele, Finland). To estimate energy expenditure during the activities (see below) we used indirect calorimetry. Expired gas samples were collected per activity via a standard mouthpiece and two-way T-shape non-rebreathing valve (2700 series) (Hans Rudolph Inc., Kansas City, MO, USA) into a Douglas Bag (DB) (Plysu Industrial Ltd., Milton Keynes, UK). Expired gas sample concentrations of oxygen and carbon dioxide inside the DB were determined using a Servomex 5200 gas analyser (Servomex Group Ltd., Crowborough, UK). The gas analyser was calibrated prior to each participant’s testing session. The total volume of expired gas inside the DB was analysed using a calibrated dry gas meter (Harvard Apparatus Ltd., Edenbridge, UK).

### Laboratory-based activity protocol

Participants were asked to perform ten laboratory-based activities of daily living which were assumed to be representative for older adults. Half of the participants (N = 20, 50% female) were instructed to arrive in a fasting condition, allowing to drink water up to a maximum of 250 ml only, while the other half received no instructions. The protocol started with 20 minutes rest in a supine position. Then, the following ten standardised activities of daily living (four minutes each) were executed in the specified order: 1) lying supine on a treatment bed, 2) sitting on a chair, 3) standing upright, 4) shuffling sideways, 5) free over-ground walking at self-selected speed, 6) cycling on an ergometer at a preferred pace (Monark Exercise AB, Vansbro, Sweden), 7) treadmill walking at 3.2 km∙h^-1^, 8) treadmill walking at self-selected speed, 9) treadmill walking at self-selected speed wearing a weighted vest (15% of body mass) and 10) brisk treadmill walking at a maximum speed of 6.5 km∙h^-1^. All treadmill walking was performed on a slat-belt treadmill (Woodway USA Inc., Waukesha, WI, USA). The first two minutes of each activity were used to reach a steady state in EE. During the second half of the activities, two one-minute expired gas samples were taken. To prevent any carry-over effects of fatigue, participants were seated between the activities until their heart rate returned to resting level. The total duration of the protocol was approximately 90 minutes. A standard digital video camera was time-synchronised and used to record the entire testing session, which served as a criterion measure and allowed direct observation of all activities post laboratory protocol completion.

### Accelerometer data pre-processing & feature selection

Analysis of the triaxial accelerometer data required multiple steps. Firstly, raw acceleration signals per axis were filtered twice using a zero-phase fourth order low pass Butterworth filter: 1) a cut-off frequency of 20 Hz was applied to remove any noise and 2) a cut-off frequency of 0.5 Hz was used to split the noise-filtered signal into static and dynamic acceleration signals, allowing determination of monitor orientation and movement [6,37]. Secondly, two one-minute periods (identical to the gas sampling minutes) of both static and dynamic acceleration signals per axis were extracted per performed activity. Next, twenty time- and frequency domain based features per non-overlapping 10-s windows were determined per axis for each of the samples extracted from both the dynamic and static acceleration signals. These time- and frequency domain based features included: arithmetic mean, standard deviation (SD), minimum, maximum, median, interquartile range (IQR), skewness, kurtosis, root mean square, cross-correlation, roll, pitch, yaw, peak-to-peak amplitude, peak intensity, zero-crossings, lag one autocorrelation, dominant frequency, amplitude of dominant frequency and entropy. Also, two resultant vectors were calculated over the three axes, one using arithmetic means and the other SDs. (Please see Liu et al. [38] for the applied formulas.) All data pre-processing was done using R 3.2.5 [39].

After data pre-processing, the 10-s window features were used to model four algorithms based on methods using either cut-off points or machine learning. Three algorithms including posture classification (based on the 10-s window arithmetic mean static acceleration of the Y-axis (static Y_mean_)) were derived from cut-off point analyses using dynamic acceleration data. The first algorithm used the sum of vector magnitudes (SVM) as an outcome,

$$SVM=\sum_{d=1}^{600}\sqrt{x_{d}{}^{2}+y_{d}{}^{2}+z_{d}{}^{2}}$$

where d represents the data-point number within the 10-s window. The second algorithm used summation of the time integrals of the moduli of the triaxial accelerometer signal (IMA), where

$$IMA=\int_{t=t_{0}}^{t_{0}+T}\left|x\right|dt+\int_{t=t_{0}}^{t_{0}+T}\left|y\right|dt+\int_{t=t_{0}}^{t_{0}+T}\left|z\right|dt$$

where T represents 10 seconds. The last cut-off point algorithm was adapted from our previous postural balance studies that focus on total movement (TM) using force plate balancing tasks [40], which is calculated as

$$TM=\sqrt{{x_{SD}}^{2}+{y_{SD}}^{2}+{z_{SD}}^{2}}$$

where SD represents the 10-s window standard deviation of the dynamic acceleration signal per axis. For the only machine learning algorithm we used Random Forest in this study, which is known for its high performance [24,41–43]. Briefly, Random Forest is an ensemble method using the bootstrapping of multiple decision trees to predict an outcome. Prior to developing a Random Forest model, analyses were performed to select optimal features for the Random Forest classifier. Firstly, pairwise correlations between features were studied, removing either one of the factors when *r* >0.75, then feature selection was performed in R 3.2.5 [39] using the Boruta package [44]. Eventually, 55 features were selected for the Random Forest model.

### Activity intensity classification

To classify activity intensities, we used metabolic equivalent (MET) values. These values were calculated per participant for all the one-minute expired gas samples taken during the activity protocol. Due to individual differences, this was done by dividing the VO_2_ (in ml∙kg^-1^∙min^-1^) during a one-minute activity sample by the participant’s calculated REE. Thus,

$$MET_{1\;min\;act\;sample}=\frac{{VO}_{2\_1\;\text{min}\;act\;sample}}{{REE}_{participant}}$$

Intensity classification for each one-minute sample (6 x 10-s windows) was done by checking 1) the MET value and 2) the participant’s posture using the video recording. Practically, when the one-minute sample’s MET value was ≤1.5, the laboratory-based activity was classified as either sedentary activity or standing, depending on the posture. Classification of light-intensity PA (LIPA) and moderate-to-vigorous PA (MVPA) was based on the MET value only, meaning if >1.5 and <3 then an epoch was classified as LIPA, while epochs with MET values ≥3 were classified as moderate-to-vigorous PA (MVPA) [10]. Intensity classification of the laboratory-based activities per this system represented the reference classification used for algorithm development and cross-validation.

### Algorithm development and cross-validation

The initial step in cut-off point based algorithm development was to create a scatterplot in MS Office Excel 2016 (Microsoft Corp., Redmond, WA, USA) using the 10-s window data, with either SVM, IMA or TM values on the horizontal axis and MET values on the vertical axis. Next, trend line-analysis was performed and the line-of-best fit (i.e. showing the highest proportion of explained variance (R^2^)) was chosen. The calculated cut-off points for SVM, IMA and TM represented MET values of 1.5 and 3, which allow classification of activity intensities per 10-s windows based on SVM, IMA and TM values, either or not combined with posture detection. Briefly, these cut-off point algorithms only use two steps in their classification structure: 1) comparing SVM, IMA or TM values with the calculated cut-off points and 2) if necessary, posture detection (Table 2).

**Table 2:** Cut-off point algorithm classification scheme.

Rules		*Classification*
1	If MET value ≤1.5 and not upright, then:	Sedentary
2	Else: If MET value ≤1.5 and upright, then:	Standing
3	Else: If MET value >1.5 and <3, then:	LIPA
4	Else: MET value ≥3, then:	MVPA

MET, metabolic equivalent; LIPA, light-intensity physical activity; MVPA, moderate-to-vigorous physical activity.

**Fig 1:**
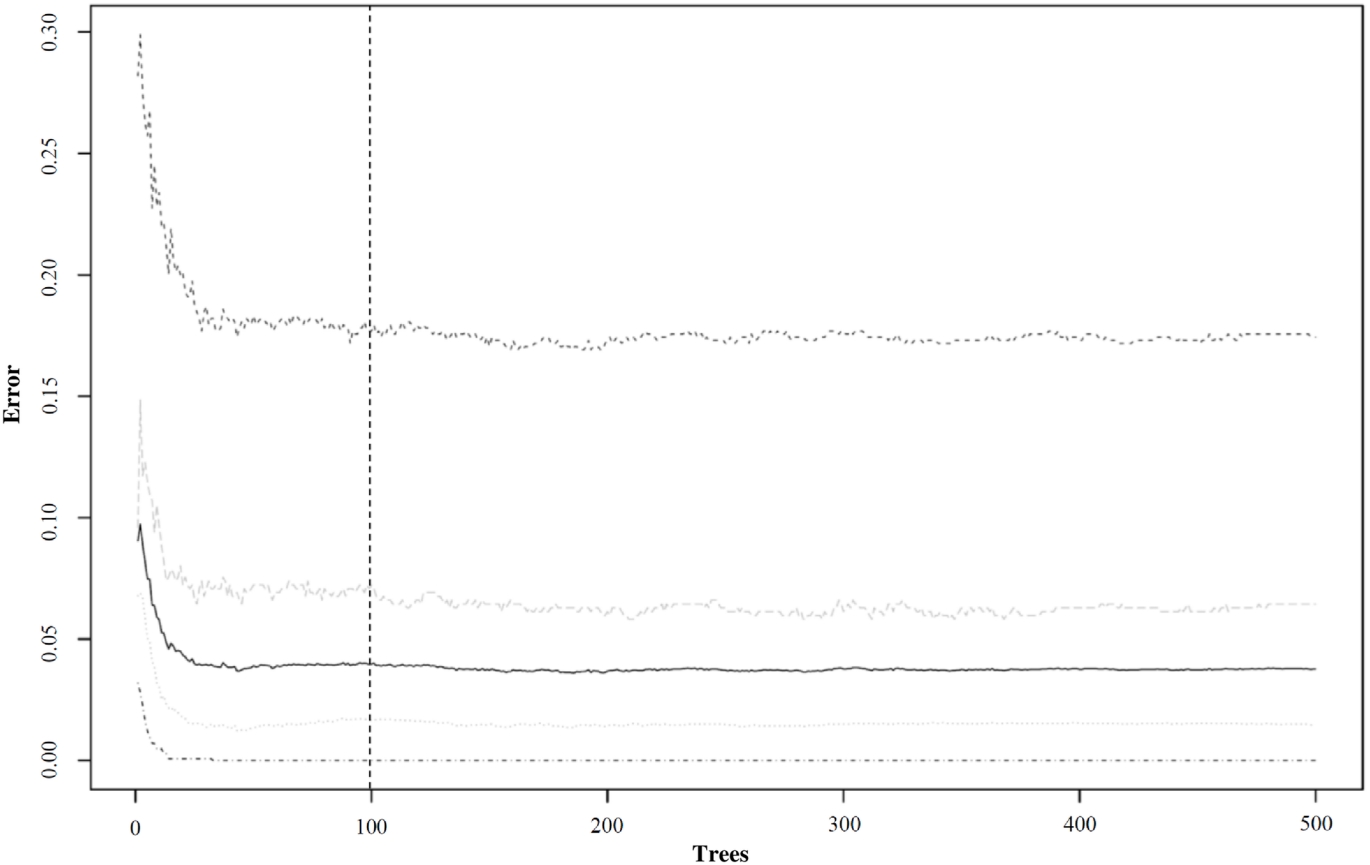
Out-of-bag error analyses for Random Forest modelling.

Random Forest model development on 10-s window features was performed in R 3.2.5 [39] using the randomForest package [45]. The 10-s window reference classifications of the laboratory-based activities were used to train the Random Forest classifier (supervised machine learning) with the number of trees set to 100. This number was derived from out-of-bag error analyses (Fig 1).

For this study, pooled-data algorithms were developed using the leave-one-subject-out method. This means that the 10-s window data of N = 39 (training sample; on average 1427 (8.6) data points for SB, 620 (7.4) for standing, 761 (19.9) for LIPA and 2937 (35.5) for MVPA) was used to develop the pooled-data algorithms, while the data of N = 1 was used to cross-validate the algorithms. With N = 40 this cross-validation procedure was repeated 40 times with another participant to be left out each iteration. Based on the performed 10-s window cross-validations, confusion matrices were created per participant per algorithm. Eventually, these matrices were used to determine balanced accuracy per intensity for each algorithm from two perspectives: 1) participant-specific and 2) overall (all participants’ confusion matrices summed).

$$Balanced\;accuracy\;(\%)=\frac{Sensitivity+Specificity}{2}$$

$$Sensitivity\;(\%)=\frac{True\;positives\;(N)}{True\;positives\;(N)+False\;negatives\;(N)}*100$$

$$Specificity\;(\%)=\frac{True\;negatives\;(N)}{True\;negatives\;(N)+False\;positives\;(N)}*100$$

where N represents the number of cases. Apart from the cross-validation, all algorithms were also tested on their own training samples to check for overfitting. Balanced accuracies of ≥80% were considered of an acceptable level [2].

### Statistical analyses

Prior to summarising or testing data, we checked its distribution for normality. Since we had a data sample of N = 40, the Shapiro-Wilk test was used for this purpose. Baseline characteristics are presented as the arithmetic mean (SD) (or median (IQR)). To test robustness of the four pooled-data algorithms we assessed if continuous baseline characteristics were correlated with balanced accuracy values (either Pearson or Spearman correlation). Differences in balanced accuracy values between categories of categorical baseline characteristics were tested with the independent T-test (or Mann-Whitney U test). For the comparison between the four pooled-data algorithms the one-way ANOVA repeated-measures test (or the Friedman test) was performed. Balanced accuracy levels from these analyses are reported as arithmetic mean (95%-confidence interval (95%-CI) (or median (~95%-CI)). In case multiple comparisons were necessary for hypothesis testing, either Bonferroni or Sidak correction was used to adjust P-values.

$$Adjusted\;P-value_{Bonferroni}=P_{value}*k$$

$$Adjusted\;P-value_{Sidak}=1-{\left(1-P_{value}\right)}^{k}$$

where k is the number of comparisons. For the current study, P-values were considered statistically significant when P <0.05.

With data variability, even within-subject under controlled conditions, and variance being one of the components for algorithm prediction errors, detailed data reliability checks were deemed highly important. Since 24 × 10-s windows bilateral accelerometer data and two one-minute expired gas samples were collected per laboratory-based activity, reliability of both main triaxial accelerometer (static Y_mean_, SVM, IMA & TM) and oxygen consumption data was determined by calculating a coefficient of variation (CV) per activity per participant.

$$CV\;(\%)=\frac{SD_{activity/participant}}{{Arithmetic\;mean}_{activity/participant}}*100$$

where SD represents standard deviation. To check for consistency across the activity protocol, all CVs were checked for correlation with MET values. If a correlation was found, data dispersion was determined (SD or IQR). Finally, depending on the distribution, either the arithmetic mean (95%-CI) or median (~95%-CI) was calculated over the moduli of all CVs per outcome variable to get sample-based reliability measures. In this study, a CV of <10% was considered acceptable.

All statistical analyses were executed using IBM SPSS Statistics for Windows, version 23.0 (IBM Corp., Armonk, NY, USA).

## Results

### Data reliability

Relationships with MET values were only found for the CVs of accelerometer outcomes SVM and static Y_mean_, ρ -0.105 (P = 0.046) and ρ -0.382 (P<0.001) respectively. IQRs for these variables were between 3.4% and 8.5% (SVM), and between 0.4% and 2.1% (static Y_mean_). The sample-based CVs of static Y_mean_, SVM, IMA and TM were 0.8% (0.7%, 1.0%), 5.5% (5.1%, 6.0%), 5.6% (5.2%, 6.2%) and 6.2% (5.7%, 7.0%) respectively. CVs of oxygen consumption data collected using the DB method also showed a negative relationship (ρ -0.495 (P<0.001)) with MET values. As shown by the IQR, VO_2_ CVs were typically between 2.2% and 7.5%. The sample-based CV of the DB method was 4.4% (3.4%, 5.3%). For all variables, the CVs within the IQR were <10%.

### Overall balanced accuracy

The confusion matrix shows that all algorithms classified sedentary activity with overall balanced accuracies of ≥99.5% (Table 3). Sensitivity and specificity values were ≥99.2%.

**Table 3:** Algorithm cross-validation confusion matrix.

Cross-validation									Individual results	Training sample
Method	Intensity	Reference				Sensitivity (%)	Specificity (%)	Balanced accuracy (%)	Acceptable level (%)	Balanced accuracy (%)
		Sedentary	Standing	LIPA	MVPA					
SVM	Sedentary	**1463**	0	12	0	99.9	99.7	99.8	100.0	99.8
	Standing	0	**588**	48	0	92.5	99.1	95.8	92.5	95.8
	LIPA	1	48	**448**	61	57.4	97.8	77.6	62.5	78.0
	MVPA	0	0	272	**2951**	98.0	90.6	94.3	100.0	94.4
IMA	Sedentary	**1463**	0	12	0	99.9	99.7	99.8	100.0	99.8
	Standing	0	**588**	48	0	92.5	99.1	95.8	92.5	95.8
	LIPA	1	48	**469**	66	60.1	97.8	78.9	65.0	79.2
	MVPA	0	0	251	**2946**	97.8	91.3	94.5	100.0	94.6
TM	Sedentary	**1454**	0	12	0	99.3	99.7	99.5	100.0	99.5
	Standing	0	**588**	48	0	92.5	99.1	95.8	92.5	95.8
	LIPA	10	47	**398**	67	51.0	97.6	74.3	57.5	74.5
	MVPA	0	1	322	**2945**	97.8	88.8	93.3	100.0	93.3
Random Forest	Sedentary	**1463**	0	34	0	99.9	99.2	99.6	100.0	100.0
	Standing	0	**585**	48	0	92.0	99.1	95.5	92.5	100.0
	LIPA	1	47	**497**	82	63.7	97.5	80.6	80.0	100.0
	MVPA	0	4	201	**2930**	97.3	92.9	95.1	100.0	100.0

SVM, sum of vector magnitudes; IMA, integrals of the moduli of acceleration signals; TM, total movement; LIPA, light-intensity physical activity; MVPA, moderate-to-vigorous physical activity.

Classification of standing was ≥95.5% accurate in all four models. Sensitivity was 92.5% in the cut-off point algorithms and 92.0% for Random Forest, while specificity was equal over the four algorithms (99.1%).

Most variation in overall balanced accuracies was found for LIPA, ranging from 74.3% (TM) to 80.6% (Random Forest). The confusion matrix revealed that the models’ sensitivity was only 57.4%, 60.1%, 51.0% and 63.7%, for SVM, IMA, TM and Random Forest respectively. On the other hand, specificity values were ≥97.5% for all algorithms.

Finally, overall balanced accuracies of ≥93.3% were found for MVPA classification. Sensitivity was ≥97.3% in all models, while specificity varied from 88.8% (TM) to 92.9% (Random Forest).

The overall balanced accuracies per intensity per algorithm were comparable between the cross-validation and training sample, except for Random Forest (Table 3). Standing, LIPA and MVPA showed overall balanced accuracies of 100.0% on the training sample against 95.5%, 80.6% and 95.1% during cross-validation.

### Robustness

Random Forest was the only algorithm not showing any changes or differences in balanced accuracies per intensity for all individual’s baseline characteristics. The cut-off point algorithms did show changes for a single baseline characteristic each, namely body height. More specifically, balanced accuracies for standing were positively correlated with body height (all three algorithms ρ 0.392 (P = 0.047)).

### Algorithm comparison

**Fig 2:**
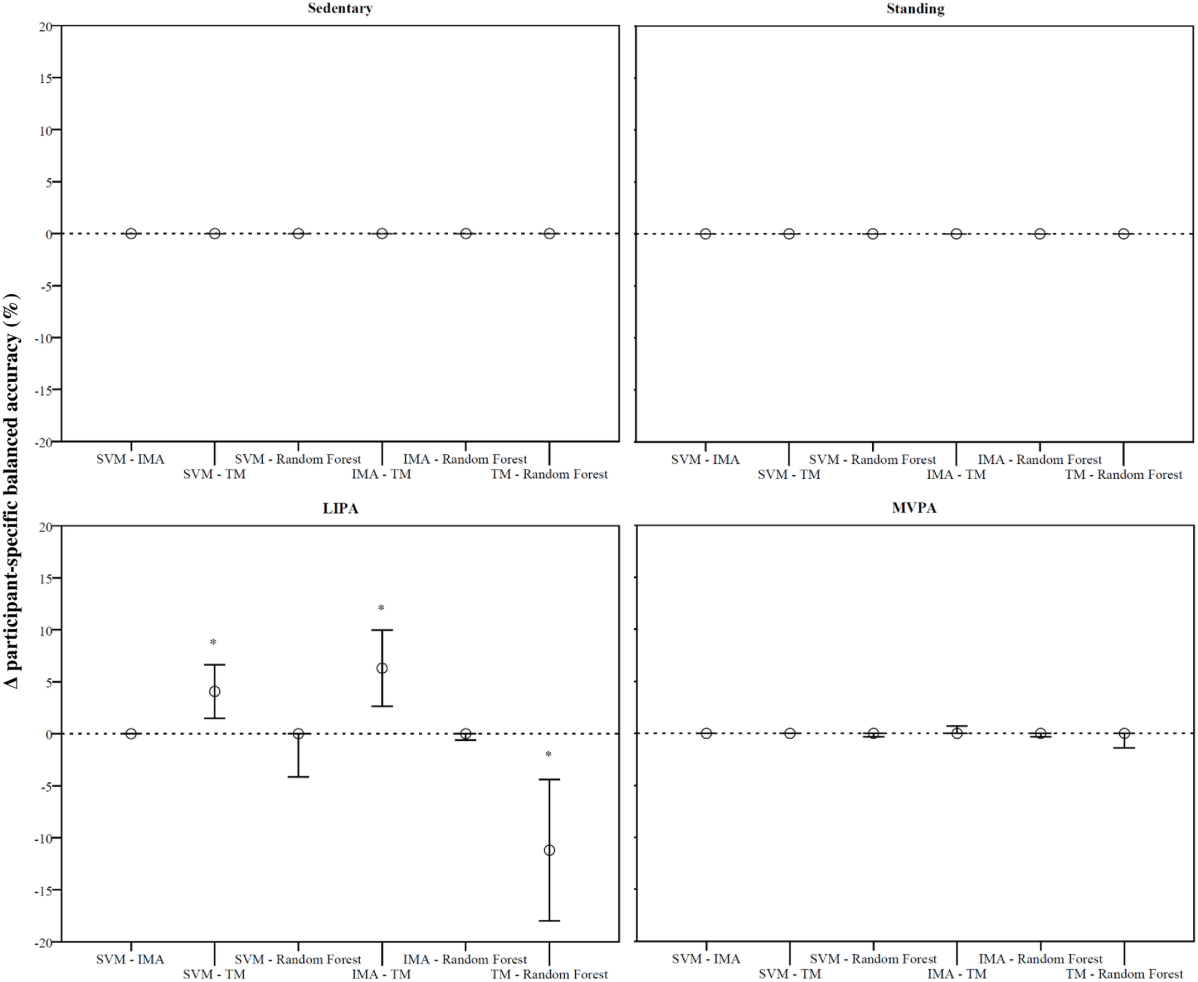
Pairwise comparisons between algorithms per intensity using participant-specific balanced accuracies. SVM, sum of vector magnitudes; IMA, integrals of the moduli of acceleration signals; TM, total movement; LIPA, light-intensity physical activity; MVPA, moderate-to-vigorous physical activity; Error bars represent 95%-confidence intervals; Dashed line represents no difference; *P <0.05.

Overall, differences in participant-specific balanced accuracies between algorithms were found for one intensity only (Fig 2). More specifically, participant-specific balanced accuracies for LIPA classification were different in three occasions, where SVM, IMA & Random Forest appeared superior over TM. The differences found were 4.1% (1.5%, 6.6%) (P = 0.006), 6.3% (2.6%, 10.0%) (P<0.001) and -11.2% (-18.0%, -4.4%) (P = 0.030) respectively.

### Algorithm benchmarking

Applying the critical 80%-threshold to the overall balanced accuracies of the pooled-data algorithms per intensity showed that all algorithms reached the threshold for sedentary activity, standing and MVPA classification (Table 3). However, only the Random Forest model also met the criterion for LIPA classification.

Benchmarking the participant-specific balanced accuracies per intensity for each algorithm revealed that all models had a perfect score (100.0%) for sedentary activity and MVPA (Table 3). The balanced accuracy for standing classification was acceptable for 92.5% of the participants in all algorithms. LIPA classification, however, showed acceptable balanced accuracies for only 62.5% (SVM), 65.0% (IMA) and 57.5% (TM) of the participants in the cut-off point algorithms, while this was 80.0% in Random Forest.

## Discussion

The main aim of the current paper was to compare between traditional cut-off points algorithms and a machine learning approach, to provide the best performing heterogeneous pooled-data algorithm to study SB and PA in older adults using thigh-mounted triaxial accelerometry. It is encouraging to note that all models showed acceptable overall balanced accuracies for classification of sedentary activity, standing and MVPA. As hypothesised however, Random Forest outperformed the cut-off point classifiers, being robust for all individual’s physiological and non-physiological characteristics and the only algorithm with acceptable (≥80%) overall balanced accuracies over the whole range of activity intensities. In addition, participant-specific balanced accuracies of Random Forest were superior over TM when classifying LIPA.

The fact that Random Forest algorithm performance was better than cut-off point models of SB and PA intensity detection is likely owing to its ability to recognise patterns in non-linear and complex data by using a combination of multiple decision trees, each trained on a random set of features [6,30]. To illustrate the difference with cut-off point algorithms, these models were developed using only two parameters from the triaxial accelerometer data, whereas modelling of the Random Forest algorithm used 55 parameters. Despite this, the differences in performance found between the cut-off point algorithms and Random Forest were rather small only. When comparing balanced accuracies between the cut-off point algorithms tested, an explanation for the results might come from the variability of the parameters used to develop the algorithms. Since oxygen consumption data was used similarly for all models, this parameter did not result in any differences. Nevertheless, with a CV of 4.4% (3.4%, 5.3%), DB proved to be a reliable method in the current study. The fact that all algorithms used the same parameter for posture detection, static Y_mean_ respectively, means that it can also be ruled out as a possible explanation for algorithm performance differences. With a CV of only 0.8% (0.7%, 1.0%) in this study, this parameter was considered highly reliable. Based on the balanced accuracies, TM is the lowest performing algorithm showing either similar or inferior balanced accuracy results per intensity when compared to the other cut-off-point algorithms. Although the CV of TM as a parameter is only 6.2% (5.7%, 7.0%), it is slightly higher than the CVs of SVM and IMA, 5.5% (5.1%, 6.0%) and 5.6% (5.2%, 6.2%) respectively. The use of a parameter representing dataset dispersion (the SD in TM), rather than a summation or integration of all data points may well be the explanation for comparatively poorer performance. As reflected by their CVs, SVM and IMA are equally performing classifiers. Although not all parameter CVs showed consistency with increasing MET values, the CVs within the IQR of all parameters were of an acceptable level (<10%), which might have resulted in acceptable overall balanced accuracies (≥80%) for all intensities of the cut-off point algorithms, except LIPA. Generally, when looking at the overall balanced accuracies per cut-off point algorithm, a similar pattern can be discovered. Sedentary activity and standing are the most accurately classified intensities, then MVPA and ultimately LIPA. The main issue with LIPA classification, for as well cut-off point algorithms as Random Forest, is the poor sensitivity (51.0%–63.7%), which is predominantly caused by misclassification with MVPA. Since the MET value range for LIPA classification is relatively small compared to MVPA’s, the LIPA/MVPA threshold is easily surpassed and therefore any amount of movement is more likely to be classified as MVPA instead of LIPA.

The positive relationships found between balanced accuracies and body height for standing classification in all three cut-off point algorithms during robustness analyses, may be due to another reason than body height. Although we standardised accelerometer mounting position by using 50% of the femur length, absolute measures show different positions, which could affect accelerometer signals. Namely, the distance to the centre of rotation (hip and knee joint respectively) influences accelerometer measurements proportionally [46]. For identical movements, the larger the distance to the centre of rotation (as in taller people), the greater the dynamic acceleration compared to that measured at positions closer to the centre of rotation (as in smaller people). This over-registration of dynamic acceleration could lead to false classification of activities with higher intensities instead. Looking at the confusion matrices, standing does show lower sensitivity values for the cut-off point algorithms, which results from misclassification with LIPA. Altogether, this implies that taller people would have lower balanced accuracies than smaller people, but frankly, we found positive correlations. Moreover, we only saw the robustness issues for standing and no other intensities. Therefore, it is plausible to assume that it was not body height to cause any changes in balanced accuracies of standing for the cut-off point algorithms. Further analysis showed that there were only three people with considerably lower balanced accuracies for standing (75% vs. ≥96.2%). Interestingly, they were amongst the smallest study participants (≤1.60 m). In addition, the confusion matrices showed that all the standing misclassifications happened in these three participants, while ten others of ≤1.60 m body height showed balanced accuracies like taller participants. Hence, when leaving the three out of the correlation analyses, no significant relationships between balanced accuracies of cut-off point algorithms for standing classification and body height were found anymore. When looking into more detail at the raw data, we noticed that the misclassifications in fact occurred during sideways shuffling, for which the three involved participants also happened to exhibit EE ≤1.5 MET. As a result of the latter, the reference classification for this activity was standing but the algorithms classified it as LIPA due to motion sensing. Thus, it was not the ‘body height’ parameter, which negatively affected the algorithm robustness results in these rare cases. Therefore, it is safe to say that all algorithms in this current study are robust, which is most probably the result of using a heterogeneous study sample.

Whilst it was encouraging to note that all algorithms showed acceptable overall balanced accuracies for classification of sedentary activity, standing and MVPA, Random Forest was the only model that also achieved the critical 80%-threshold for LIPA classification. Despite the generally good results, the disadvantage of an overall measure is that it can mask unacceptable algorithm performance on an individual basis. For that reason, it is also important to check the percentage of acceptable participant-specific balanced accuracy per intensity for each model. This revealed that individual classification of sedentary activity and MVPA was always of an acceptable level, which allows categorisation of people based on the amount of SB and MVPA, such as active, inactive and active couch potato. Moreover, standing classification was acceptable for 92.5% of the participants in all algorithms. On the contrary, LIPA classification was acceptable in only ≤65.0% of the participants when using a cut-off point algorithm, while this number rose to 80.0% in case Random Forest was used. To summarise, these results show that the cut-off point algorithms presented in the current study, can be used to detect SB, standing and MVPA in older adults confidently. The Random Forest algorithm, however, can be used for the same outcomes, including LIPA classification too. This latter is exciting, because LIPA might play an important role in gaining health benefits by counteracting SB through PA in elderly [1]. Moreover, performance of MVPA may have negative physiological effects, such as increased inflammation, and not necessarily elicit any greater physiological benefits over LIPA in the older adult population [47]. Additionally, performing MVPA may have a high threshold as well as poor long-term adherence in elderly.

Compared to recent research that, similarly to our present one, conducted laboratory-based testing to validate activity intensity identification algorithms including machine learning, our results are in fact a further improvement on these classifiers because we also focus on algorithm robustness and benchmark individual accuracies [2,23,48]. Although comparing results between studies is complicated by differences in populations, monitor placement (mainly hip or wrist, against us thigh) that may influence classification [2], and outcome variables (e.g. Kappa statistic vs. balanced accuracy) [42], our overall finding is in agreement with Ellis et al. [6]. They also showed improved free-living activity intensity classification with machine learning over traditional cut-off point models (without posture detection). However, it must be noted that their machine learning algorithm was developed using free-living accelerometer data only, while the traditional cut-off points were derived in the laboratory.

One could consider the development of algorithms under laboratory conditions as a limitation, given the fact that when laboratory-based, performance during real-life mobility monitoring is compromised [2,6]. However, in the laboratory, conditions can be controlled and a whole range of activities and intensities can be studied allowing calibration, while simultaneously providing proof-of-concept such as thigh-mounted triaxial accelerometry in older adults [2,24]. To improve the matching of performance from laboratory-based with free-living based accelerometer algorithms one may match the amount of data collected on each behaviour with its prevalence in free-living and train the algorithms with bout lengths similar to true daily life behaviour [24]. Although our use of steady-state data of activities with predefined length will improve algorithm accuracies [2], this may not be directly translated to data collected outside the laboratory, since steady-state is not necessarily reached in free-living conditions with activities being more sporadic [24]. Also, Gyllensten and Bonomi [49] found that activities in free-living conditions exhibit a higher degree of overlapping characteristics in their acceleration features when compared with activities performed in the laboratory. Some free-living activities even show substantially different acceleration signals in comparison to when performed in the laboratory [2,24]. Although we agree that true performance of our algorithms in real-life conditions cannot necessarily be derived from the balanced accuracies seen under laboratory settings and it will probably be lower in free-living, we do not expect the dramatic decrease (~13%–46%) reported elsewhere [2,6,24,48,49]. There are several reasons supporting this expectation. Firstly, most of these studies are either not comparable to our study in terms of study population, modelling techniques/settings, extracted features, and accelerometer placement, or suffered from serious methodological issues such as using the same sample to both develop and validate algorithms [2,6,24,48,49]. Secondly, we included few, but common basic activities for elderly persons in our protocol [50–52], and instructed participants to perform them as ‘naturally as possible’ i.e. using self-selected speed and/or intensity. Next, instead of activity classification, we used intensity classification (based on individual REE corrected MET values) in our study, which is a more generic system providing less options, and thus expected to be less prone to error when applied outside the laboratory [24]. Finally, we used a heterogeneous sample, representing the true healthy older adult population, to develop the algorithms.

Another potential study limitation may be the fact that our models have been developed for application in a single thigh-mounted accelerometer, which does not allow perfect monitoring of PA, as perhaps wobbling of thigh mass or the lack of upper-body movement detection results in classification errors [10,27]. Although it has been suggested that mounting multiple sensors could address the latter issue [10,27,53], study compliance may become compromised [48], something that is less of a problem with a single accelerometer [21,27]. Moreover, thigh mounting can accurately distinguish between sitting and standing, which is not possible with traditional monitor placement at the hip or waist [19,20,54,55]. This placement is thus superior to detect upright stationary activities common in the household, that tend to be more metabolically demanding than activities that recruit only the upper body. Thigh mounting is also relatively close to the centre of mass, which is vital for good prediction of EE and monitoring of locomotion [10,16]. Capturing locomotion is important in elderly, because it provides information about physical independence [10]. Generally, a combination between thigh-mounted accelerometry and machine learning is considered ideal, because the latter in fact makes sensor placement less relevant [27].

The major strength of our current approach is that its design and protocol are largely in accordance with the recommendations for accelerometry-based studies done by Welk et al. [32]. To highlight these compelling elements, despite being modestly sized (~16.4 hrs of algorithm training data only), a study sample containing a large variety of physiological and non-physiological characteristics was used to develop four different accelerometer algorithms. The analyses were performed in more detail (such as focusing on robustness and benchmarking individual accuracies) than usually seen in the literature. The use of leave-one-subject-out cross-validation, ideal for smaller datasets, minimises the risk of overfitting with Random Forest machine learning and enhances the general applicability of the algorithms to new data [56]. Additionally, by using a reliable method for measuring oxygen consumption (CV 4.4% (5.3%)) and correcting for individual metabolic baselines, coupled with direct observation, the reference intensity classification is highly accurate. Since both raw accelerometer data and videos were collected, post-study analyses will be possible such as algorithm tuning, epoch length optimisation or activity classification, but also comparisons with other monitors. Most importantly, this is the first study to conduct detailed analyses of heterogeneous pooled-data algorithms, ranging from simple cut-off point to complex machine learning, for the quantification of SB and PA in older adults using thigh-mounted triaxial accelerometry.

Future studies should focus on further analysis and development of the Random Forest algorithm to classify activities qualitatively. This will not only result in better prediction of EE [57], but also provide information not captured by intensity classification [4,6,24]. Moreover, the Random Forest algorithm should be validated in a free-living set-up and compared to a similar algorithm developed on free-living data. Furthermore, comparisons with proprietary algorithms of commercially available activity monitors would be interesting, not least to allow direct comparison of data from different laboratories and hence the creation of large data sets. Overall, these suggestions would 1) improve understanding of the associations between human activity and health that will inform future recommendations and guidelines for older adults to support healthy ageing [4,6,24] and 2) help to improve current industry standards in activity monitoring in elderly.

## Conclusions

Unlike the cut-off point algorithms, under laboratory conditions the Random Forest machine learning model showed acceptable algorithm performance throughout the whole range of activity intensities in older adults wearing a thigh-mounted triaxial accelerometer. Its performance of LIPA classification in particular, makes the algorithm highly relevant for this age group. The fact that this pattern recognition technique 1) does not require subgroup-specific calibrations and/or specific accelerometer body part positioning, 2) is capable of recognising actual human activities and 3) works independent of accelerometer brand/settings, signifies its potential large-scale applicability to distinguish SB and different levels/types of PA in older adults.
